# Hospital and Institutional News

**Published:** 1918-05-04

**Authors:** 


### HOSPITAL AND INSTITUTIONAL NEWS.

### NEW CHIEF COMMISSIONER OF CANADIAN RED CROSS

Lieut.-Colonel H. W. Blaycock, who since 1915 has held the rank of Assistant Commissioner at Boulogne, has been appointed by the Canadian Red Cross Society Chief Commissioner Overseas. Colonel Blaycock, a Canadian by birth, who has lived in England since 1908, has been mentioned in dispatches by Sir Douglas Haig and received the Legion of Honour. He is an Associate of the Order of St. John of Jerusalem.

### FULHAM PALACE AS A HOSPITAL.

In consequence of the offer of Fulham Palace by the Bishop of London to the British Red Cross Society six weeks ago, the Freemasons' War Hospital Committee is opening it as a military hospital. Mrs. M. Fox-Symons has been appointed matron, and Sir Horace Marshall is treasurer. The Palace will add one hundred beds to those previously provided by the craft at its hospitals at Chelsea and Caversham. The War Office has approved the arrangements, and of course the patients are not limited to Freemasons. Apart from its historic interest, the Palace is known to many people from the Bishop's monster garden-parties which used to be held there in summer. On those occasions those who were considerate and curious used to stand aside and watch the long procession of visitors approach the Bishop, who, leaning at intervals on a chair, used to shake hands in turn with an apparently endless line of waiting guests. The enjoyment of the afternoon would not have been complete to many if that handshake had been missed; but the ordeal was a long one to the Bishop.

### THE SOUTH AFRICAN HOSPITAL.

The South African Hospital in Richmond Park, since 1915, which now possesses 653 beds, is appealing for £35,000, a sum which represents a two years' excess of expenditure, in spite of the support of the South African Red Cross. The fund which has provided the hospital has many claims upon it. It has contributed to the South African Ambulance at Cannes, and supplied comforts to the South African Base Hospital in France. It started and equipped the South African Officers' Club in Grosvenor Square, and attends to the wants of prisoners, to men on leave, and to detachments on arriving from South Africa. Discharged men waiting for their passage home also come under the care of the fund. Lord Gladstone, therefore, has not hesitated to appeal for help to all with South African connections.

### POOLING APPEALS AT SHEFFIELD.

The financial straits of the Sheffield Royal Hospital and the Sheffield Royal Infirmary have led the committees of the two institutions to issue a joint appeal for £100,000. In this way competition will be avoided, and the bigger end in view and the double organisation are believed likely to ensure success. Hospital indebtedness at Sheffield is an unusually old story, and the fact is worth mentioning because the hospital of which Mr. J. W. Robinson is secretary has received nearly 500 soldiers without any charge to the Government. The cost of this national work is put down at nearly £9,000. The total indebtedness of this hospital is equal to twice its annual income. It is hoped that the workmen will agree to contribute more than one penny per week. But an appeal should take the form of an organised plan for employers as well as employed, by which the former should be invited to contribute a fixed sum per head of every person in their employment. The class from which annual subscriptions are drawn should also have its own organisation. So should the outlying districts which send patients to the two hospitals. By these means, and these only, can the financial future of the two institutions be made secure.

### THE WEAKNESS OF VIGILANCE SOCIETIES.

The National Council of Public Morals, one of those unofficial bodies which requires watching in consequence of the general tendency of such societies to make a fetish of repressing innocent things, has accomplished two useful services. It has caused diachylon, a favourite abortifacient, to be placed upon the Schedule of Poisons, and it has published a Report on the Birth-rate containing much useful information on the voluntary sterilisation of marriage which is now general. It has also busily advocated the establishment of a Ministry of Health, and is now extending the scope of its Birth-rate Commission to investigate the economics of parenthood, the housing problem, the industrial employment of women of child-bearing age, and similar questions. Its publication department is equally busy, and its inquiry into the influence of the cinema has led it to propose a State censorship. All experience is against the success of such a scheme, which is popular because repression is popular and seems to offer an easy solution. Official censors, however, never know chalk from cheese, invariably repress the good and countenance the evil. The proper remedy is prosecution after performance, as is the current method of dealing with objectionable books. It is noteworthy that councils of this type and with such titles usually end in making repression into a method for its own sake, and are apt to become the prey of the crudest faddists. For this reason such bodies are even more in need of criticism than of praise for the good which they accomplish incidentally.

### THE HOSPITAL LOOK.

In describing the aim and work of Kitchener House, the club for wounded soldiers, where classes are given and occupations are taught, Mr. John Galsworthy, in the *Times*, lately referred to the lethargic "hospital look" which the club had been founded to remedy. He pointed out the "apathy," "the look of a mind inevitably divested of mental initiative," which, he said, were encouraged by "long spells of hospital life." We know it well, but we think that he overstated his case in limiting this apathy to hospital life only. Ask any intelligent soldier, and he will tell you that the worst part of military life, of the daily routine of the Army, is precisely the blank hours of waiting, of compulsory loafing in fact, with nothing to do and nowhere to go, except to hang about the camp. Military life encourages this apathy, it is part of the system, and the intolerable *ennui* which is thus produced is the worst hardship which an educated man has to bear. The number of vacant hours imposed on men by Army life is really appalling, and no doubt the tradition of them descends to the hospitals too. But the origin is not in them; the worst part is not their share: the root of the mischief is in the Army itself. How far clubs for soldiers, who are not wounded, could provide relief from this boredom and waste of time is uncertain. But certainly, apart from active service, the Army produces more boredom than any other institution yet invented. The evil persists in hospital, but its source is in the barracks and the camp.

### THE "IMPERTINENCE" OF THE LONDON COUNTY COUNCIL.

Mr. Rooth, the magistrate at the Thames Police Court, referred in an indignant manner to the inaction of the London County Council in the case of a young man who was mentally deficient. Since the passing of the Act which placed these unfortunate people under the charge of the London County Council where local Boards of Guardians have declined to accommodate them, the London County Council has been at a loss to know what to do with the patients. In the case before Mr. Rooth the young man had been previously before the Court and remanded, and the same procedure was again requested. Mr. Rooth was indignant. "This poor wretched fellow," he said, "has been remanded again and again, and the London County Council forgets he is a human being. The London County Council has treated the Court with scant courtesy, for it has not thought fit to answer letters, although, at the last possible moment, the telephone was used. Correspondence should be conducted by the ordinary means, and what the Council has done is a great piece of impertinence. The unfortunate fellow is being detained in gaol awaiting the pleasure of this great authority." Until the London County Council succeeds in finding accommodation for mentally deficient persons such criticism as Mr. Rooth's is inevitable, and will be repeated.

### TURN OF FORTUNE'S WHEEL.

A pleasing instance of gratitude, showing how a commercial man's early struggles are remembered in prosperity, has been reported to the governors of the Leicester Royal Infirmary by Colonel C. J. Bond, C.M.G., the deputy-chairman and consulting surgeon. The incident cannot be better described than in the donor's own words:—"Dr. C. J. Bond. Sir,—About twenty-five years ago my wife, since deceased, was an inmate of the Leicester Infirmary, and I made up my mind then that, if ever I lived to be able to, I would repay in some measure, the great care and kindness she received there, and especially the skill and attention which you yourself devoted to her case, to which, I am sure, the saving of her life was due. I am taking the liberty of sending you £100, which I shall be glad if you will use for the benefit of the Royal Infirmary in any way you deem best. I feel it is my duty to tell you that, although my wife lived until 1917, she never forgot your kindness and devotion." The present gift recalls a similar incident when a few weeks since, on the occasion of the visit of the "Tank" to Leicester, a father and mother called at the infirmary to ask the institution to accept a War Bond for £100, which the turn of fortune's wheel in their favour, as they expressed it, enabled them to offer in gratitude for the successful treatment of their son fifteen years ago by a present member of the honorary surgical staff. Incidents such as these are gratifying testimony to the regard in which the voluntary hospitals are held. It should appeal to successful business men, if the productivity of their manufactures can be maintained at the present war standard.

### THE MINT AND THE RED CROSS.

To raise money for the Red Cross and to increase the reserve of gold and silver in the Bank, a collection of gold and silver in any form is being organised. A committee has been formed, and the Mint has arranged to buy the metal from the Red Cross. Gold and silver of every kind, ornaments, plate, medals, and even odds and ends will be welcome. The address of the office to which communications should be addressed is 39 Old Bond Street.

### TRINKETS AND APPEALS.

The Royal Sussex County Hospital is to benefit by a collection of similar articles to those mentioned in the previous paragraph, which will either be sold or else melted. The scheme originated with two friends of the hospital, who hope to enlist the assistance of the Pound Day collectors, and thereby to secure the remaining £4,000 which must be raised if the hospital's recent appeal for £10,000 is to be successful. As the war continues we are variously reminded that there are hardly any waste products, and, from soot to old artificial teeth, everything has a use, and consequently a market.

### EDUCATIONAL LECTURES TO SOLDIERS.

In connection with the Great Northern Central Hospital, a lecture on "Florence the Home of the Renaissance" was lately given by Mr. B. C. Hastwell, Head of the Art Department, L.C.C. Shoreditch Technical Institute, in the Military Annexe, Manor Gardens. A set of slides selected from the collection at the South Kensington Museum was shown, and an interesting account of the city, its surroundings, and art treasures was given. Arnolfo di Cambio's work on the Duomo, Brunelleschi's Dome, the Campanile, etc., were dealt with by the speaker, whose personal reminiscences of visits to the city gave vitality to the lecture. The results of the close relation of arts and crafts as evidenced by the work of Giotti, Donatello, Cellini, the Della Robbias, and others of the old craftsmen gave us reasons for sympathy with our own William Morris and the work of such bodies as the Arts and Crafts Guild, the Society of Art and Industry, etc. The Medici and the other merchant princes of Florence, Savonarola, Fra Angelico, and Fra Bartolommeo are world renowned. After pointing out some of the differences between Northern Gothic and Classic architecture, between the craftsmanship on various gates and other details, and explaining why the city rose to such eminence in the fourteenth and fifteenth centuries, Mr. Hastwell gave the soldiers some advice on books, recommending for their study Ruskin's "Mornings in Florence," Mrs. Oliphant's "Makers of Florence," George Eliot's "Romola," and the writings of John Addington Symonds.

### STAFF VACANCIES AT NOTTINGHAM.

We regret to record the resignation of Miss Gertrude Knight, who has been matron of the Nottingham General Hospital for over twenty-five years. Since the war the hospital's accommodation has been doubled, and the last years of Miss Knight's matronship have probably been the most arduous of all. The appointment of her successor is announced on another page. The hospital is also losing for a time the services of Mr. E. M. Keely, who has held the post of secretary for thirty-two years. Mr. Keely has never recovered from the accident which befell him last October, and his health, which has been failing, necessitates a rest. At this time important negotiations are on foot for the purchase of a site between the hospital and the castle from the trustees of the late Sir Charles Seely for future extensions after the war.

### EXTENSION TO QUEEN MARY'S HOSPITAL.

A memorial to the West Ham men who have fallen in the war is being undertaken by Mr. W. Thorne, M.P., Mayor of West Ham. It will take the form of an extension to Queen Mary's Hospital for the East End, formerly known as the West Ham Hospital. Mr. Thorne hopes to raise a sum of £30,000. At present 100 beds are being used for wounded soldiers.

### A MEDICAL SUPERINTENDENT'S QUARTERS.

Dr. Graham, resident medical superintendent of the Belfast City and District Asylum, has complained that his present apartments are wholly unsuitable, and that his occupation of them is not conducive to discipline. The late medical superintendent occupied Purdysburn House, which is now about to be used for patients. Dr. Graham is therefore temporarily accommodated in apartments, and though he knew of this at the time of his appointment, he has said that he deferred his objections until experience proved to him the inadequacy of the arrangements. A committee had previously gone into the matter, which has, however, been raised again—not only by Dr. Graham—and we do not think that the last has been heard of it.

### A POTATO PATCH FOR A HOSPITAL.

An unexpected gift of a somewhat novel kind has come the way of King Edward VII.'s Hospital, Cardiff, in the shape of over a ton of potatoes, sent all the way from Portrush, in Ireland, by Mr. J. S. Chubb, an old Cardiff man, and delivered through the kindness of Messrs. Spillers and Bakers' Shipping Department. Furthermore, Mr. Chubb has written to the secretary of the hospital: "I may state that I am planting a patch of potatoes for you which I am calling the King Edward VII. Patch, and drills of carrots and parsnips to be known as King Edward VII. Drills." Gifts in kind on these lines open up quite fresh possibilities, and no doubt other good-hearted people may be inspired by Mr. Chubb's example to go and do likewise.

### THE DISABLED AS DENTAL MECHANICS.

The Manchester Dental Hospital has made arrangements to train from twelve to twenty disabled sailors and soldiers as dental mechanics. A competent instructor and assistant have been engaged, and after the twelve months to which the course extends are over, it is expected that the men instructed will be able to earn from £3 to £4 per week. The chairman of the governors, Mr. Tom Wilson, in lately appealing for increased financial aid, appeared to be hopeful of the new scheme, the results of which will be watched with interest.

### A SUBJECT FOR RESEARCH.

The latest hospital to instal a cinematograph appears to be Cardiff Mental Hospital, since the ladies' committee entrusted with the supervision of the comforts for the wounded has given the cinematograph and the necessary platform and equipment. On the agreement of the committee to let the cinematograph remain in the hospital after the war, the board of control has accepted it. We hope not only that the men will have some fun from it, but that the board will conduct a little research into its possibilities, since we do not believe that either commercially or educationally the moving film has been developed to anything like the limit of its capacity. It can represent an accident. It can also represent the growth of a plant. In each case by judicious compression. These well-known powers should be sufficient to induce Lieut.-Col. Edwin Goodall, who, if we mistake not, is fond of experimental research, to turn his mind to the problem of what can be done to make a cinematograph worthy of its place in hospital life.

### DAY SANATORIA FOR LONDON CHILDREN.

In connection with the comprehensive scheme for the treatment of tuberculosis in London, the County Council has had under consideration the establishment of day sanatoria for children in association with the Borough Councils. Inquiries show that many children suffering from tuberculosis would gain considerable benefit from attendance at day sanatoria, where treatment and education would be given. In a few boroughs arrangements have been initiated by voluntary committees in conjunction with the dispensaries, and the County Council has arranged for the supply of teachers. The Council thinks that the establishment of such sanatoria might be regarded as part of the work of the dispensaries. In communicating these views to the Borough Councils, the County Council mentions that the Local Government Board would regard these day sanatoria as part of the dispensary organisation, and pay a grant of 50 per cent. of any expenditure incurred by the Borough Councils.

### THE MILDMAY MISSION HOSPITAL.

The Mildmay Mission Hospital, in Bethnal Green, which was reopened last May after thorough reconstruction of the drainage system, has ended the year well with a balance of £850 to the good. The Mildmay Institutions, comprising the Memorial and the Mission Hospitals, the Nurses' Home, and the Conference Hall and Deaconess House, have lately been entirely reorganised; the Conference Hall and the Deaconess House have passed into the hands of the Young Men's Christian Association, and each of the medical charities is now carried out under separate management, the Mildmay Trustees merely holding the property. The missionary character of the work is still maintained, services being held in the wards and out-patients' department, while Bible-classes for women and children are held in the Mission Hall. We regret to note that the reopening of the Children's Ward has been deferred owing to the shortage of the nursing staff.

### IS THE VOTE WORTH THE DISCLOSURE?

The "Representation of the People Act, 1918," which accords the vote to women over the age of thirty, imposes upon secretaries the task of ascertaining which of their nurses and domestics have attained to what is apparently, in Parliamentary opinion, the feminine age of discretion. Some secretaries will no doubt adopt the inglorious plan of passing "Form A" on to matron for completion, while others will place the women in the position of disclosing their ages, and (or?) losing their votes. The secretary of a London hospital lately posted the following notices in the staff dining-rooms: "Under the Representation of the People Act, 1918, the franchise is accorded to women above a certain age. The secretary is instructed to make a return to the Registration Officer in order that the new register may be completed. Members of the staff who desire their names entered, if eligible, should consult the secretary on or before the date mentioned."

### NEWS FROM BRITISH PRISONERS.

The April number of the *British Prisoner of War* is something of a literary curiosity, for it contains certain "Lines Written in Captivity" by Captain F. S. Patmore, 5th Hampshire Regiment, the son of Coventry Patmore, a great and neglected name in English poetry. Curiously enough, Captain Patmore has happened to choose the metre in which his father's poem "The Falcon" was written, a poem, it should be added, which was written in a metre quite unsuited to his genius. The long straggling line with six beats, in which the two poems are written, is more suitable to rhetoric than to poetry, and it has rarely been handled with success except by rhetorical writers. Captain Patmore, however, writes sincerely: his vision of home and of India is clearly remembered, and his lines have a distinction of their own, which transcends the interest of their authorship. Soon we hope to give an illustrated account of the new trades at which British prisoners interned in Switzerland are being employed. Of much general interest is the account of a walk through Treves by a British officer, which is reprinted from the Treves officers' journal called the *Barb*. It describes the privilege of leaving the barbed-wire camp for a walk, a privilege which resulted from "a long-standing arrangement between England and Germany entered into through the American Ambassador at Berlin, before America joined the Allies." There are also some notes from prisoners in Turkey, and many points of news or suggestions for those who are anxious to send parcels to prisoners.

### FIRST WOMAN CANDIDATE FOR THE PHARMACEUTICAL COUNCIL.

For the first time in the history of the Pharmaceutical Society of Great Britain a woman pharmacist is a candidate for a seat on the Council. The election will take place in May, and is likely to be keenly contested. There are twelve persons nominated for seven seats. Miss Buchanan, the woman candidate, was one of the first women to take the pharmaceutical qualification, and is also a silver medallist. She is now teacher of pharmacy and dispensing at the Royal Free Hospital Medical School. Miss Buchanan was the founder of the Association of Women Pharmacists, a small but growing organisation. Many women, not content with obtaining a "certificate in dispensing," are now taking the full qualification of the pharmacist, which confers on them the statutory right to carry on the business of chemist and druggist and to go on the panel of chemists under the National Health Insurance Act. It would appear only natural that they should have a direct representative on the Pharmaceutical Council. With the general body of women pharmacists interested in the result of the election will be many medical women who have received tuition in dispensing and pharmacy from Miss Buchanan at the Royal Free Hospital.

### TO OUR READERS.

**Contributions are specially invited from any of our readers to these columns. They should deal with topical subjects and news. They must be authenticated for the information of the Editor only. The minimum payment if published is 5s. There is no hard-and-fast rule as to space, but notes of about twenty lines in length are preferred.**

